# Copulation defective mutants of *C. elegans*

**DOI:** 10.17912/W2XH3S

**Published:** 2017-11-02

**Authors:** Yvonne M. Hajdu-Cronin, Katharine S. Liu, Leslie Barber, Helen M. Chamberlin, William Boorstein, Paul W. Sternberg

**Affiliations:** 1 Division of Biology and Biological Engineering, Caltech, Pasadena CA 91125; 2 Department of Molecular Genetics, Ohio State University, Columbus, OH 43210

## Description

To identify genes involved in male copulatory behavior, we carried out an F2 clonal screen in a *him-5* mutant background. We identified 20 mutations that affect male mating behavior without causing gross defects in morphology.

Male mating in *C. elegans* comprises at least five steps (Liu and Sternberg, 1995). (l) The male responds to the hermaphrodite by backing his tail along the length of the hermaphrodite, (2) he turns over or under her body before reaching the head or tail, (3) he locates the vulva with his tail, at which point he stops backing, (4) he inserts his spicules into the vulva, and (5) he transfers sperm. To study the genetic basis for male mating behavior, we are isolating and characterizing Copulation Defective (Cod) mutations. We screened for mutant strains defective in this behavior using the screen described by Hodgkin (1983). *him-5(e1490)* worms are mutagenized with ethyl methane sulfonate (EMS); 20 P0 L4 hermaphrodites are picked singly to Petri plates; ten F1 worms are picked per mutagenized P0; and ten F2 L4 hermaphrodites are singled per P0 and their male progeny tested for mating efficiency via a qualitative mating test (six males crossed with six *unc-52(e444)*hermaphrodites, which are paralyzed at adulthood (Brenner, 1974). Mutants with phenotypes that are likely to reduce mating efficiency in a non-specific manner (such as those causing an Unc, Dpy, or Lon phenotype) were discarded. Those strains that appear morphologically normal under the dissecting microscope yet fail to mate or mate at a very low efficiency (1-5% cross progeny compared to wild type) were screened under Nomarski optics for defects in male reproductive structures. We screened over 3000 haploid genomes, and picked over 100 strains with reproduction defects. Nineteen strains were successfully backcrossed, which represents about 25% of the total strains attempted. This result suggests that most strains harbor two or more mutations that contribute to the mating-deficiency defect. Preliminary analysis of behavior suggests that every major step in the wild-type mating pathway has at least one corresponding Cod mutation blocking the behavior, with several mutations blocking at the spicule insertion step. The screen also yielded morphological mutants, whose phenotypes include crumpled spicules, abnormal rays, and a gonad migration defect; some of these will be described elsewhere (Chamberlin & Sternberg; micropublication in preparation).

**Table 1. Mating-defective alleles from the Cod screen.** Gene assignments are from data that will be presented elsewhere. Phenotypes are from the preliminary analysis carried out as part of the secondary screens; some will be described in more detail later.

**Table:** 

**Allele**	**gene**	**phenotype**
*sy155*		Response defective (step 1)
*sy166*	*cod-3*	Response defective (step 1)
*sy172*	*che-3*	Response defective (step 1)
*sy178*		Response defective; Uncoordinated (step 1)
*sy35*	*cod-11*	Turning (step 2)
*sy174*		Turning, vulval location (steps 2,3)
*sy181*	*cod-5*	turning, vulval location (steps 2,3)
*sy157*		vulval location (steps 3)
*sy38*	*cod-10*	Spicule insertion (step 4)
*sy43*	*cod-2*	Spicule insertion (step 4)
*sy153*		Spicule insertion (step 4)
*sy156*		Spicule insertion (step 4)
*sy158*		Spicule insertion (step 4)
*sy165*		Spicule insertion (step 4)
*sy176*	*cod-8*	Spicule insertion; synthetic with sy226 (step 4)
*sy226*	*cod-9*	Synthetic with sy176 (step 4)
*sy177*		Spicule insertion (step 4)
*sy36*		Few progeny
*sy179*		Few progeny
*sy180*	*cod-4*	Few progeny

## Reagents

**Strains:**

**PS611:** *sy155;* *him-5**(**e1490**)*

**PS708:** *cod-3(sy166);* *him-5**(**e1490**)*

**PS947:** *vab-2(e96) cod-3(sy166);* *him-5**(**e1490**)*

**PS1040:** *cod-3(sy166) dpy-4(e1166);* *him-5**(**e1490**)*

**PS1000:** *cod-3(sy166);* *him-5**(**e1490**)*

**PS1001:** *dpy-13(e184) cod-3(sy166);* *him-5**(**e1490**)*

**PS685:** *che-3(sy172);* *him-5**(**e1490**)*

**PS701:** *che-3(sy172); dpy-17(e164);* *him-5**(**e1490**)*

**PS992:** *che-3(sy172);* *him-5**(**e1490**)*

**PS854:** *sy178; **him-5**(**e1490**)*

**PS995:** *sy35;* *him-5**(**e1490**)*

**PS1781:** *sy35; dpy-4(e1166);* *him-5**(**e1490**)*

**PS140:** *sy35;* *him-5**(**e1490**)*

**PS705:** *sy174;* *him-5**(**e1490**)*

**PS862:** *sy174; unc-4(e120);* *him-5**(**e1490**)*

**PS948:** *dpy-5(e61); sy174;* *him-5**(**e1490**)*

**PS872:** *cod-5(sy181);* *him-5**(**e1490**)*

**PS997:** *cod-5(sy181);* *him-5**(**e1490**)*

**PS1140:** *cod-5(sy181); dpy-4(e1166);* *him-5**(**e1490**)*

**PS1768:** *cod-5(sy181) rol-1(e91);* *him-5**(**e1490**)*

**PS2836:** *cod-5(sy181); dpy-20(e1282);* *him-5**(**e1490**)*

**PS613:** *cod-?(sy157);* *him-5**(**e1490**)*

**PS996:** *cod-2(sy43);* *him-5**(**e1490**)*

**PS1139:** *cod-2 (sy43); dpy-17(e164);* *him-5**(**e1490**)*

**PS148:** *cod-2(sy43);* *him-5**(**e1490**)*

**PS609:** *sy153;* *him-5**(**e1490**)*

**PS991:** *sy156;* *him-5**(**e1490**)*

**PS1094:** *dpy-13(e184) (sy156);* *him-5**(**e1490**)*

**PS612:** *sy156;* *him-5**(**e1490**)*

**PS1141:** (*sy158); dpy-11(e224)* *him-5**(**e1490**)*

**PS614:** *sy158;* *him-5**(**e1490**)*

**PS707:** *sy165;* *him-5**(**e1490**)*

**PS711:** *cod-8(sy176); cod-9(sy226);* *him-5**(**e1490**)*

**PS939:** *cod-8(sy176); dpy-13(e184) cod-9(sy226);* *him-5**(**e1490**)*

**PS981:** *cod-8(sy176); cod-9(sy226);* *him-5**(**e1490**)*

**PS988:** *cod-8(sy176); cod-9(sy226); dpy-11(e224)* *him-5**(**e1490**)*

**PS1782:** *cod-8(sy176); cod-9(sy226);* *him-5**(**e1490**)*

**PS876:** *sy177;* *him-5**(**e1490**)*

**PS141:** *him-5**(**e1490**); sy36*

**PS874:** *sy179; dpy-4(e1166);* *him-5**(**e1490**)*

**PS887:** *cod-4(sy180); dpy-4(e1166);* *him-5**(**e1490**)*

**PS987:** *cod-4(sy180);* *him-5**(**e1490**)*

**PS1292:** *dpy-17(e164) cod-4(sy180);* *him-5**(**e1490**)*

**PS990:** *cod-10(sy38);* *him-5**(**e1490**)*
